# Triglyceride glucose index predicts all-cause mortality in oldest-old patients with acute coronary syndrome and diabetes mellitus

**DOI:** 10.1186/s12877-023-03788-3

**Published:** 2023-02-06

**Authors:** Jian Shen, Bin Feng, Li Fan, Yang Jiao, Ying Li, Henan Liu, Xiaoling Hou, Yongkang Su, Dongyun Li, Zhenhong Fu

**Affiliations:** 1 Senior Department of Cardiology, the Sixth Medical Center, Chinese PLA General Hospital and Chinese PLA Medical School, 100853 Beijing, China; 2 Outpatient Department of Tongzhou Retired Cadres Rest Center, Beijing, 101149 China; 3 Department of Nutrition, Children’s Hospital of Chongqing Medical University, Chongqing, 400014 China; 4 Department of the First Health Care, the Second Medical Center, Chinese PLA General Hospital, Beijing, 100853 China

**Keywords:** Triglyceride-glucose index, Oldest-old patients, Diabetes mellitus, Acute coronary syndrome, All-cause mortality

## Abstract

**Background:**

Acute coronary syndrome (ACS) and diabetes mellitus (DM) are the leading health risks for the elderly. Triglyceride-glucose (TyG) index is a novel and reliable indicator of insulin resistance (IR). This study aims to explore the relationship between the TyG index and all-cause mortality in oldest-old patients with ACS and DM.

**Methods:**

Seven hundred twenty hospitalized patients with ACS aged ≥ 80 years were enrolled, and 699 patients signed informed consent for the study. During the follow-up period, 37 were lost to follow-up, and the follow-up rate was 94.7%. 231 ACS patients with DM were selected for the study’s analyses. Kaplan–Meier curve, Cox regression model and receiver operating characteristic (ROC) curve were used to analyze the association between the TyG index and all-cause mortality.

**Results:**

The mean age of participants was 81.58 ± 1.93 years, and 32.47% were women. Compared to TyG tertile 1, the Hazard Ratio (HR) [95% confidence interval (CI)] of all-cause mortality was 2.04 (1.09, 3.81) for TyG tertile 3 in the fully adjusted model. For the TyG index per standard deviation (SD) increment, the HR (95% CI) of all-cause mortality was 1.44 (1.13, 1.83). Further, the association between the TyG index and all-cause mortality was dose–response (*P* for trend = 0.026). ROC curve analyses indicated that the TyG index outperformed FBG and TG in the prediction of mortality risk and improved the prognostic value of the Gensini score combined with LVEF.

**Conclusion:**

The TyG index predicts the risk of all-cause mortality in the oldest-old ACS patients with DM.

## Introduction

With the acceleration of population aging, the population of the elderly is dramatically increasing [1, 2]. DM and coronary heart disease (CHD) are common chronic diseases that jeopardize seniors’ health [3]. The latest data shows that cardiovascular disease remains the leading cause of mortality worldwide [4]. It is well known that DM is one of the traditional risk factors for CHD, which could contribute to its progression and deterioration. ACS, one clinical subtype of CHD, typically represents an emergency and serious condition suggesting an increased risk of adverse cardiovascular outcomes. It is estimated that more than 37% of ACS patients have DM [5]. Compared to ACS patients without DM, those with DM are prone to worse cardiovascular events [6, 7]. In addition, DM is associated with an increased risk of mortality in ACS patients [8, 9].

The condition that insulin target tissues are weakly sensitive to metabolic reactions related to insulin is defined as insulin resistance (IR). IR is a common feature of metabolic syndrome, obesity, and DM, and also one risk factor for cardiovascular disease [10, 11]. The TyG index, calculated from fasting blood glucose and triglyceride, is a new marker of IR [12]. The hyperinsulinemic-euglycemic clamp test, considered the gold standard measure of IR, has been found to correlate well with the TyG index [13, 14]. Moreover, compared with the hyperinsulinemic-euglycemic clamp test, the TyG index has the advantages of simplicity, low price, and non-invasiveness, which is more likely to be suitable for clinical practice [15].

Previous studies have shown that the TyG index is one predictor of the risk of arteriosclerosis, coronary calcification, and DM [16–18]. Furthermore, the TyG index was found to be associated with the risk of adverse cardiovascular events in CHD patients [19, 20]. However, the relationship between the TyG index and all-cause mortality remains unclear. This study was designed to examine the association between the TyG index and all-cause mortality in the oldest-old ACS patients with DM.

## Methods

### Study design and population

**Fig. 1:**
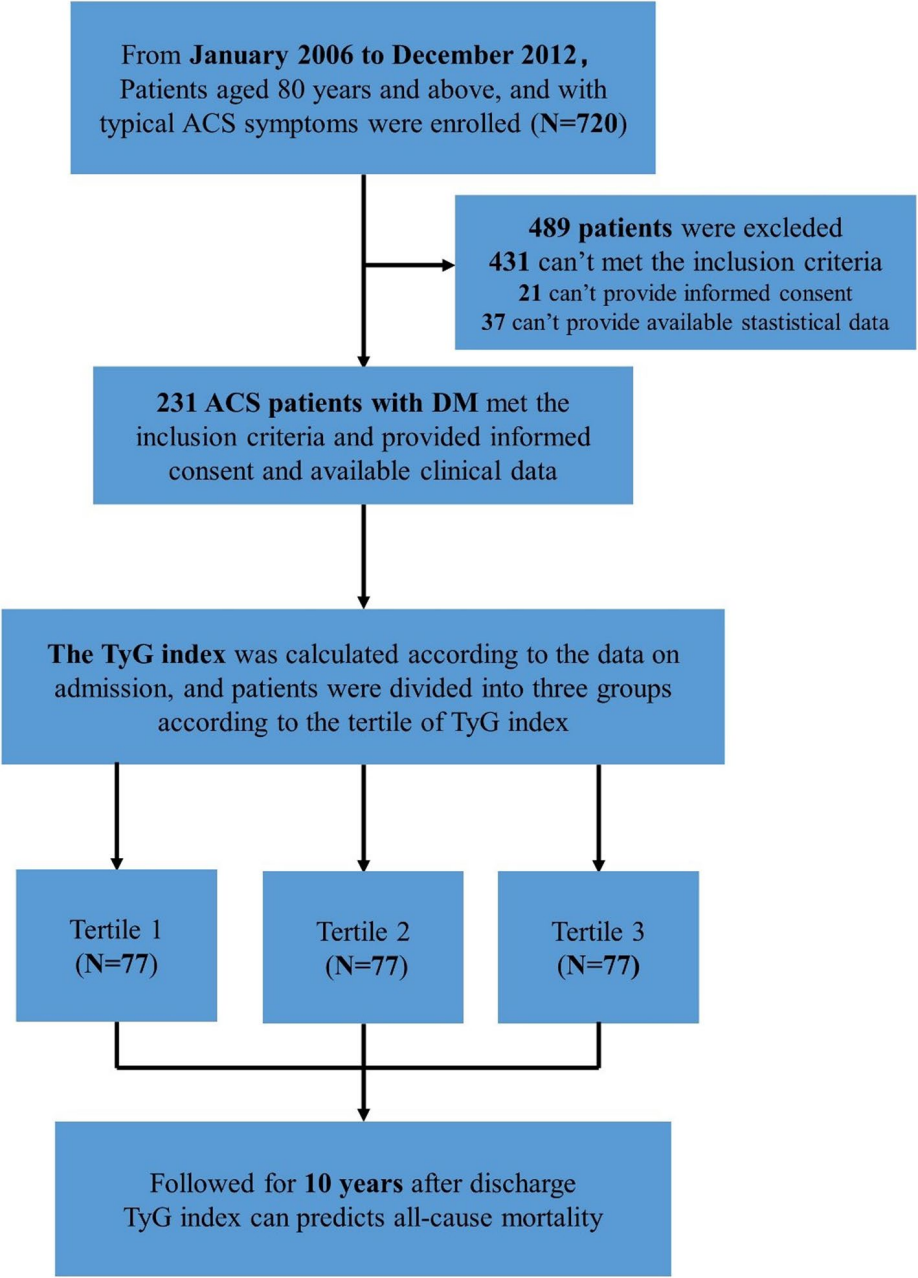
Flow chart of the study participants

From January 2006 to December 2012, all ACS patients ≥ 80 years old hospitalized in the Department of Cardiology in Chinese PLA General Hospital and who underwent coronary angiography were enrolled. The definition of ACS includes unstable angina pectoris (UAP), non-ST-segment elevation myocardial infarction (NSTEMI), and ST-segment elevation myocardial infarction (STEMI). All patients received standardized treatment based on the coronary angiography results. The exclusion criteria of this study included patients with chronic infectious diseases, types of malignant tumors, rheumatoid arthritis, severe liver insufficiency, pulmonary hypertension, severe valvular heart disease, suspected familial hypertriglyceridemia (triglyceride ≥ 5.65 mmol/L), extreme body mass index (BMI) ≥ 45 kg/m^2^ and neuropsychiatric disorders. 720 patients were recruited, and 699 signed informed consent. 37 patients were also excluded because of missing follow-up data, and the follow-up rate was 94.7%. The patients with DM were selected for the present study. DM was defined as the previous history of diabetes, fasting blood glucose ≥ 7.0 mmol/L, random blood glucose ≥ 11.1 mmol/L, or oral glucose tolerance test 2-h blood glucose ≥ 11.1 mmol/L. A total of 231 ACS patients with DM were included in the analysis (Fig. 1). This study was approved by the Ethics Service Center of Chinese PLA General Hospital and all methods were performed in accordance with the Helsinki Declaration of Human Rights.

The sample size needed for the study based on a power calculation of the number of patients required for 80% power at the P = 0.05 level, assuming approximately a 10% loss to follow up. And the number of participants we enrolled is more than the sample size we assessed. Based on the number of participants in the analyses, the Hazard Ratio and the overall probability of event, the power of the research as well as the risk of type II error were calculated.

### Data collection

The patients’ general information such as sex, age, height, weight, systolic blood pressure (SBP), diastolic blood pressure (DBP) and smoking history, past medical histories such as hypertension, hyperlipidemia, myocardial infarction (MI), chronic kidney disease (CKD) and stroke, fasting biochemical indicators such as fasting blood glucose (FBG), total cholesterol (TC), triglyceride (TG), low-density lipoprotein cholesterol (LDL-C), high-density lipoprotein cholesterol (HDL-C), creatinine and uric acid (UA)], cardiovascular medication history such as aspirin, clopidogrel, statins, beta-blockers, angiotensin-converting enzyme inhibitor (ACEI), angiotensin receptor blocker (ARB)], and left ventricular ejection fraction (LVEF) were collected.

All patients performed coronary intervention. The coronary angiography results were analyzed by the same image analysis software. Coronary culprit vessels, including the left main artery (LM), left anterior descending artery (LAD), left circumflex artery (LCX) and right coronary artery (RCA), were evaluated by the trained professors. Multivessel lesions were defined as the condition that there were two or more culprit vessels. The Gensini score was calculated to estimate the severity of coronary artery stenosis. Based on the coronary angiography results, all patients received standardized and individualized therapeutic strategies, including intensive medicine, percutaneous coronary intervention (PCI) and coronary artery bypass grafting (CABG).

The TyG index was retrospectively calculated as Ln [fasting TG (mg/dl) × fasting FBG (mg/dl)/2]. Body mass index (BMI) was defined as the body mass (in kilograms) divided by the square of the body height (in meters). The equation of estimated glomerular filtration rate (eGFR) was calculated by the Chinese modified Modification of Diet in Renal Disease: eGFR (mL/min/1.73 m^2^) = 175 × standardized creatinine (mg/dL) ^−1.234^ × age(year)^−0.179^ [× 0.79 (if female)]. An eGFR < 60 mL/min/1.73 m^2^ was considered the criterion for CKD.

### Follow-up and endpoint event

The primary endpoint was all-cause mortality. After hospital discharge, all patients were routinely followed up every 12 months. The information regarding adverse events was obtained through telephone contact with patients or their family members and ascertained from a careful review of corresponding medical records. The follow-up time reached up to 10 years. Follow-up time was recorded in months, calculated by the time of the endpoint event minus the time of discharge.

### Statistical analysis

The patients were divided into 3 groups (Tertile 1, Tertile 2 and Tertile 3) according to the TyG index tertiles. Continuous data were expressed as mean ± standard deviation (SD) or median (25th to 75th percentile), and analysis of variance (ANOVA) and the Kruskal–Wallis test were used to compare differences in baseline characteristics among the TyG index tertiles according to whether the variables were normally distributed. Categorical data were expressed as n (%), and the Chi-squared test or Fisher’s exact test was used to compare proportions between groups. The correlations between the TyG index and baseline variables were analyzed using Pearson’s correlation test.

Kaplan–Meier curves were used to describe the survival between the TyG index groups and Log-rank tests were performed. A univariate Cox regression analysis was carried out to identify predictors of mortality. The cox proportional hazards model was used to analyze the association between the TyG index and all-cause mortality. In regression analysis, the selection of covariables mainly follows the following principles: 1. the correlated factors screened out by univariable Cox regression analysis; 2. relevant factors found in previous research; 3. risk factors acknowledged widely. We used the Likelihood ratio test to assess the Cox models, and the statistical test of the proportional hazard assumption was performed. Moreover, the Schoenfeld Residuals Test and Kaplan–Meier curve were visually inspected for potential time-variant biases.

Three models were built and potential confounders were adjusted in the models. Model 1 was the unadjusted model. Model 2 was adjusted for age and sex. Model 3 was adjusted for age, sex, BMI, SBP, DBP, LVEF, Gensini score, hypertension, hyperlipidemia, previous MI, previous stroke, CKD, current smoking, TC, LDL-C, HDL-C, eGFR, UA, aspirin, clopidogrel, statin, β-blocker, ACEI/ARB, LM lesion, multivessel lesion and treatment.

The TyG index was treated as three categorical variables for the primary analysis. Trend tests were used to explore the dose–response relationship between the TyG index and all-cause mortality. The TyG index was standardized and put into the regression models to determine the relationship between the TyG per SD increment and mortality. Results were expressed as hazard ratios (HR) and 95% confidence intervals (CI).

Receiver operating characteristic (ROC) curves were used to compare the discrimination capacity of the TyG index, FBG and TG to predict mortality. To compare the discrimination of the three above indicators, harrell C-statistics, continuous net reclassification improvement (cNRI), and integrated discrimination improvement (IDI) were calculated.

The statistical software packages R (http://www.R-project.org, The R Foundation) and Empower Stats (http://www.empowerstats.com, X&Y Solutions, Inc., Boston, MA) were selected to analyze all data. A two-tailed* P* < 0.05 was regarded as statistically significant.

## Results

### Baseline characteristics of all participants

Among the 231 oldest-old patients enrolled, most patients were men (67.53%). The average age was 81.58 ± 1.93 years, and the average TyG index was 9.01 ± 0.67. According to the TyG index tertiles, the patients were divided into three groups. The higher group was more likely to be female and to have elevated HR, FBG, TC, TG, and LDL-C. There was no statistical difference in age, BMI, BP, LVEF, smoking history, eGFR, UA, history of past illness, cardiovascular medications, coronary artery lesion and treatment strategy among the TyG index groups (Table 1).

**Table 1:** Baseline characteristics of all participants according to TyG index tertiles

Variables	Total	TyG index			*P-*value
		Tertile 1	Tertile 2	Tertile 3	
N	231	77	77	77	
General information					
Age, year	81.58 ± 1.93	81.49 ± 1.69	81.77 ± 2.23	81.47 ± 1.84	0.576
Female, n (%)	75 (32.47%)	14 (18.18%)	28 (36.36%)	33 (42.86%)	0.003
Height, cm	165.41 ± 8.06	166.55 ± 8.03	165.17 ± 8.40	164.51 ± 7.71	0.276
Weight, kg	67.96 ± 11.30	68.04 ± 11.89	68.12 ± 11.20	67.71 ± 10.93	0.972
BMI, kg/m^2^	24.78 ± 3.42	24.44 ± 3.45	24.94 ± 3.62	24.96 ± 3.20	0.574
SBP, mmHg	139.19 ± 21.57	139.58 ± 18.36	143.10 ± 22.03	134.90 ± 23.49	0.060
DBP, mmHg	69.77 ± 12.06	69.64 ± 10.74	70.38 ± 12.31	69.31 ± 13.16	0.855
LVEF, %	56.28 ± 9.19	58.26 ± 7.82	55.87 ± 9.55	54.72 ± 9.84	0.051
Current smoking, n (%)	67 (29.00%)	27 (35.06%)	21 (27.27%)	19 (24.68%)	0.335
Laboratory markers					
eGFR, mL/min/1.73 m^2^	69.12 ± 20.94	72.76 ± 19.90	68.75 ± 21.27	65.85 ± 21.31	0.121
FBG, mmol/L	8.51 ± 6.06	5.92 ± 1.68	7.75 ± 2.23	11.85 ± 9.21	< 0.001
UA, umol/L	354.50 ± 200.08	361.12 ± 306.02	343.06 ± 97.01	359.33 ± 133.73	0.828
TC, mmol/L	4.03 ± 0.99	3.69 ± 0.91	4.06 ± 0.95	4.35 ± 1.01	< 0.001
TG, mmol/L	1.50 ± 0.77	0.93 ± 0.29	1.38 ± 0.32	2.21 ± 0.88	< 0.001
LDL-C, mmol/L	2.26 ± 0.85	2.00 ± 0.73	2.33 ± 0.86	2.43 ± 0.89	0.004
HDL-C, mmol/L	1.09 ± 0.36	1.19 ± 0.47	1.09 ± 0.29	0.98 ± 0.26	0.002
TyG index	9.01 ± 0.67	8.30 ± 0.32	8.99 ± 0.16	9.75 ± 0.43	< 0.001
History of Past Illness					
Hypertension, n (%)	200 (86.58%)	68 (88.31%)	65 (84.42%)	67 (87.01%)	0.770
Previous stroke, n (%)	51 (22.08%)	21 (27.27%)	17 (22.08%)	13 (16.88%)	0.299
Previous MI, n (%)	35 (15.15%)	10 (12.99%)	15 (19.48%)	10 (12.99%)	0.431
Hyperlipidemia, n (%)	56 (24.24%)	20 (25.97%)	16 (20.78%)	20 (25.97%)	0.686
CKD, n (%)	37 (16.02%)	11 (14.29%)	11 (14.29%)	15 (19.48%)	0.598
Cardiovascular medications					
Aspirin, n (%)	227 (98.27%)	75 (97.40%)	76 (98.70%)	76 (98.70%)	0.775
Clopidogrel, n (%)	220 (95.24%)	75 (97.40%)	72 (93.51%)	73 (94.81%)	0.513
Statin, n (%)	213 (92.21%)	72 (93.51%)	68 (88.31%)	73 (94.81%)	0.282
β-blocker, n (%)	154 (66.67%)	53 (68.83%)	52 (67.53%)	49 (63.64%)	0.776
ACEI/ARB, n (%)	145 (62.77%)	49 (63.64%)	48 (62.34%)	48 (62.34%)	0.982
Angiography					
LAD lesion, n (%)	195 (84.42%)	69 (89.61%)	66 (85.71%)	60 (77.92%)	0.126
LCX lesion, n (%)	150 (64.94%)	50 (64.94%)	53 (68.83%)	47 (61.04%)	0.598
RCA lesion, n (%)	169 (73.16%)	56 (72.73%)	54 (70.13%)	59 (76.62%)	0.658
LM lesion, n (%)	35 (15.15%)	9 (11.69%)	15 (19.48%)	11 (14.29%)	0.390
Multivessel lesion, n (%)	180 (77.92%)	61 (79.22%)	61 (79.22%)	58 (75.32%)	0.797
Gensini score	55.44 ± 41.43	46.55 ± 29.80	58.52 ± 43.33	61.25 ± 48.01	0.064
Treatment					0.176
Intensive medication, n (%)	79 (34.20%)	31 (40.26%)	24 (31.17%)	24 (31.17%)	
PCI	145 (62.77%)	45 (58.44%)	48 (62.34%)	52 (67.53%)	
CABG	7 (3.03%)	1 (1.30%)	5 (6.49%)	1 (1.30%)	

### The TyG index was correlated with clinical variables

**Fig. 2:**
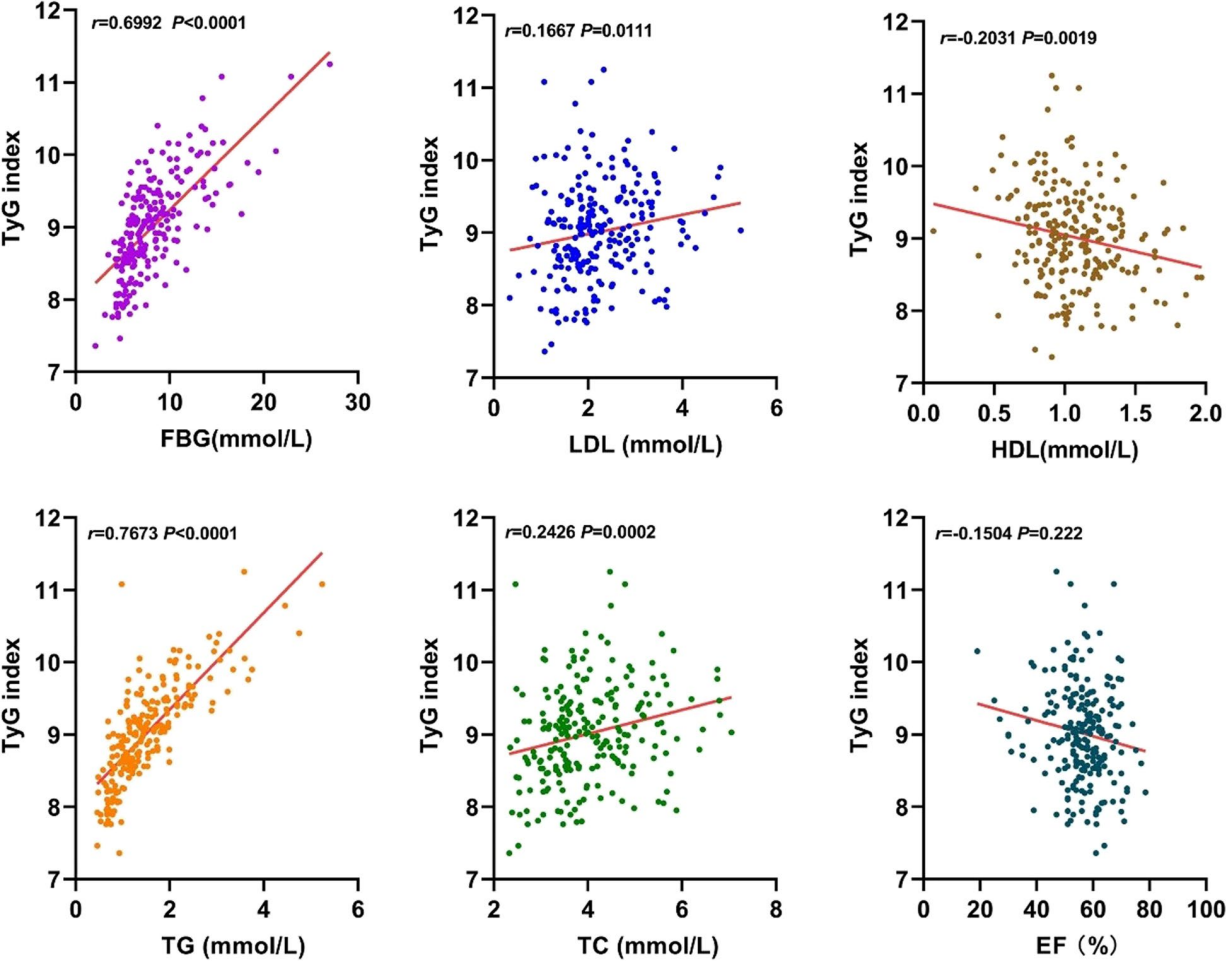
Correlation between the TyG index and clinical variables

The TyG index was positively correlated with FBG, TC, TG and LDL-C, while negatively correlated with eGFR, LVEF and HDL-C. The TyG index showed no correlation with age, BMI, SBP, DBP, UA and Gensini score. The TyG index had the highest positive and negative correlation with TG and HDL-C, respectively (Fig. 2, Table 2).

**Table 2:** Correlation between TyG index and clinical variables

Variables	Correlation coefficient, r	95%CI	*P*-value
Age	-0.049	(-0.177, 0.081)	0.458
BMI	0.039	(-0.090, 0.167)	0.554
SBP	-0.097	(-0.223, 0.033)	0.143
DBP	-0.072	(-0.199, 0.058)	0.278
eGFR	-0.135	(-0.259, -0.006)	0.041
FBG	0.556	(0.460, 0.639)	< 0.001
UA	-0.026	(-0.154, 0.104)	0.697
LVEF	-0.150	(-0.274, -0.022)	0.022
Gensini score	0.127	(-0.002, 0.252)	0.054
TC	0.242	(0.117, 0.360)	< 0.001
TG	0.767	(0.709, 0.816)	< 0.001
LDL-C	0.167	(0.038, 0.289)	0.011
HDL-C	-0.224	(-0.343, -0.097)	< 0.001

### Univariate Cox regression analysis for all-cause mortality

During a median follow-up of 49 months (interquartile range: 36 to 61 months), a total of 86 patients died. Univariate Cox regression analysis was performed to identify the potential predictors of mortality. The all-cause mortality was significantly associated with several factors, the previous stroke, CKD, HR, SBP, DBP, eGFR, FBG, LVEF, Gensini score, and the TyG index included (Table 3).

**Table 3:** Univariate Cox regression analysis for the association between baseline variables and all-cause mortality

Variables	HR (95%CI)	*P*-value
Female	1.07 (0.68, 1.69)	0.758
Age	1.11 (0.99, 1.24)	0.062
BMI	0.95 (0.90, 1.02)	0.138
Hypertension	1.17 (0.61, 2.27)	0.636
Previous stroke	1.79 (1.13, 2.83)	0.012
Previous MI	1.62 (0.96, 2.72)	0.071
Hyperlipidemia	1.02 (0.62, 1.67)	0.932
CKD	2.42 (1.49, 3.93)	0.004
Current smoking	1.06 (0.66, 1.68)	0.819
SBP	0.99 (0.98, 1.00)	0.01
DBP	0.97 (0.96, 0.99)	0.006
Aspirin	0.33 (0.10, 1.06)	0.062
Clopidogrel	0.56 (0.24, 1.29)	0.174
Statin	1.12 (0.51, 2.43)	0.780
β-blocker	1.06 (0.67, 1.66)	0.806
ACEI/ARB	1.17 (0.75, 1.84)	0.485
eGFR	0.97 (0.96, 0.98)	< 0.001
FBG	1.04 (1.02, 1.06)	< 0.001
UA	1.00 (1.00, 1.00)	0.537
LVEF	0.97 (0.95, 0.99)	0.002
Gensini score	1.01 (1.00, 1.01)	0.001
LAD lesion	0.93 (0.54, 1.60)	0.797
LCX lesion	1.28 (0.81, 2.03)	0.290
RCA lesion,	1.31 (0.79, 2.18)	0.300
LM lesion	0.98 (0.54, 1.77)	0.951
Multivessel lesion	1.67 (0.94, 2.97)	0.079
Treatment		
Intensive medication	1.0	
PCI	0.81 (0.52, 1.26)	0.343
CABG	0.26 (0.04, 1.90)	0.185
TC	1.20 (0.98, 1.48)	0.081
TG	1.26 (0.99, 1.60)	0.057
LDL-C	1.18 (0.92, 1.51)	0.204
HDL-C	0.59 (0.29, 1.19)	0.142
TyG index	1.76 (1.28, 2.42)	0.001
TyG group		
Tertile 1	1.0	
Tertile 2	1.56 (0.88, 2.78)	0.129
Tertile 3	2.13 (1.23, 3.71)	0.007

### Association between the TyG index and all-cause mortality

**Fig. 3:**
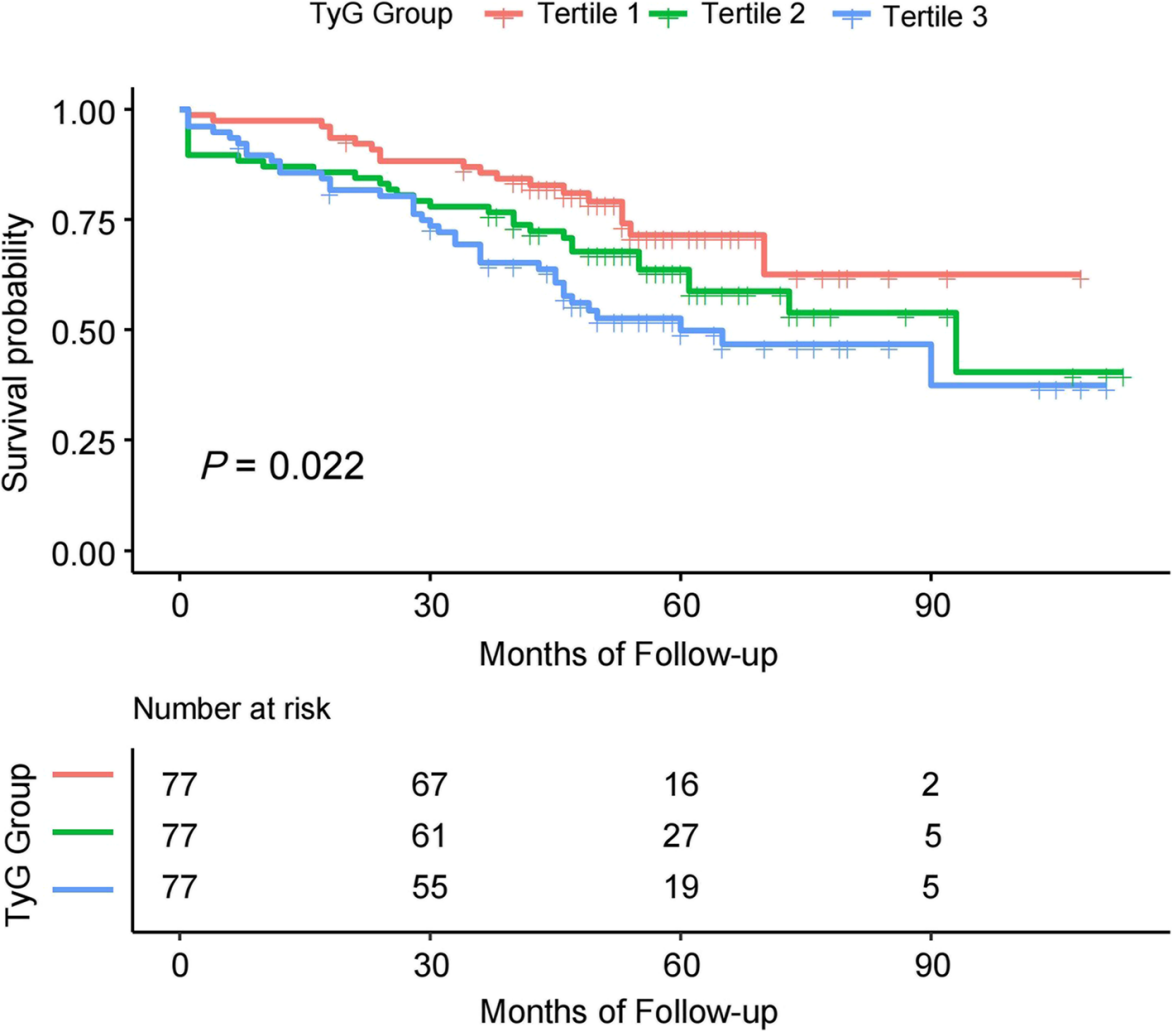
Kaplan–Meier survival curves of survival probability according to TyG tertiles

The Cox regression models were built to analyze the association between the TyG index and all-cause mortality. Based on the tertiles, the TyG index was transformed as a categorized variable in the analyses. As shown in Table 4, after fully adjusting for the confounders, the HRs (95% CIs) for all-cause mortality in Tertile 2 and Tertile 3 were 1.62 (0.87, 3.02) and 2.04 (1.09, 3.81), respectively. When the TyG index was entered as a continuous variable into the models, we found a similar result that the HRs (95% CIs) for all-cause mortality was 1.44 (1.13, 1.83) for the TyG index per SD increment. We also conducted the trend test analyses and found that there was a dose–response relationship between the TyG index and all-cause mortality (*P* for trend = 0.026). Further, the Kaplan–Meier curve showed that the patients in Tertile 3 had the worst survival probability (*P* = 0.022) (Fig. 3).

**Table 4:** Cox regression analysis for the association between TyG index and all-cause mortality

TyG index	Death, n (%)	Model 1	Model 2	Model3
Per SD increase	86(37.2)	1.46 (1.18, 1.82) ***	1.50 (1.21, 1.86) ***	1.44 (1.13, 1.83) **
TyG Tertiles				
Tertile 1	19(24.7)	1.0(Ref)	1.0(Ref)	1.0(Ref)
Tertile 2	30(39.0)	1.56 (0.88, 2.78)	1.62 (0.90, 2.89)	1.62 (0.87, 3.02)
Tertile 3	37(48.1)	2.13 (1.23, 3.71) **	2.18 (1.25, 3.82) **	2.04 (1.09, 3.81) *
*P* for trend		0.007	0.006	0.026

Model 1: unadjusted

Model 2: adjusted for age and sex

Model 3: adjusted for age, sex, BMI, SBP, DBP, LVEF, Gensini score, hypertension, hyperlipidemia, previous MI, previous stroke, CKD, current smoking, TC, LDL-C, HDL-C, eGFR, UA, aspirin, clopidogrel, statin, β-blocker, ACEI/ARB, LM lesion, multivessel lesion and treatment

^*^P < 0.05 **P < 0.01 ***P < 0.001

### Comparison of the capacity of the TyG index, FBG and TG to predict mortality

**Fig. 4:**
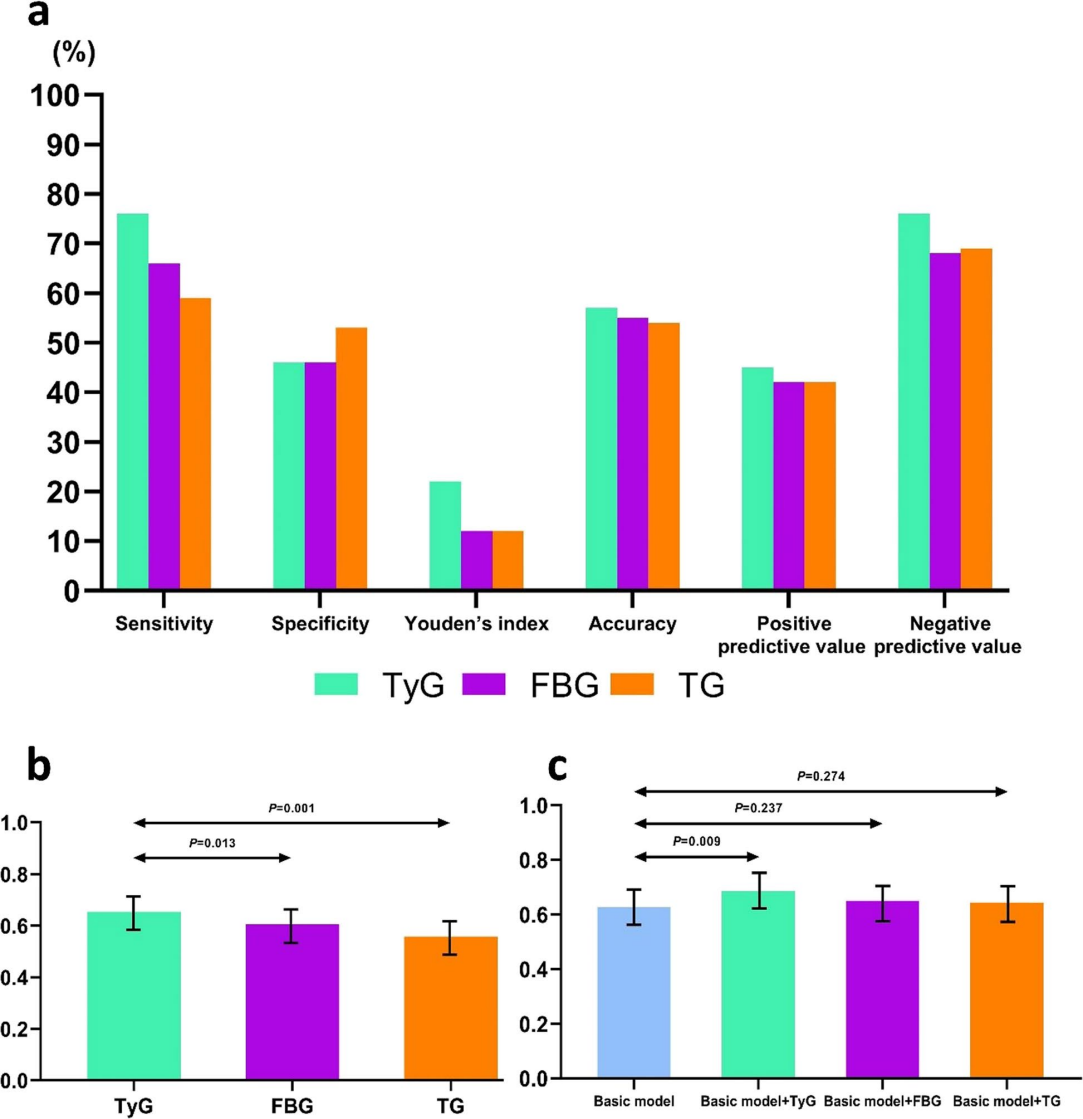
**a** Diagnostic performance of TyG, FBG, and TG for all-cause mortality; **b** C-statistic of TyG, FBG, and TG for prediction of all-cause mortality; **c** C-statistic of the basic model (Gensini score and LVEF), + TyG, + FBG, and + TG for prediction of all-cause mortality

ROC curve was used to compare the discrimination capacity of the TyG index, FBG and TG to predict mortality. The TyG index had the highest sensitivity, accuracy, positive predictive value and negative predictive value, although the specificity of the TyG index was slightly lower. Youden’s index was higher than that of FBG and TG (Fig. 4a). The C-statistics of the TyG index, FBG and TG was 0.653 (0.589–0.717), 0.606 (0.543–0.670), 0.557 (0.493–0.620) respectively. The TyG index was superior to FBG and TG at predicting all-cause mortality, as was seen by the discrimination index values(*P* < 0.05) (Table 5, Fig. 4b). For mortality risk prediction, considering the effects of traditional risk factors, the basic model on the Gensini score combined with LVEF was constructed for further research. The C-statistics of the Gensini score + LVEF + TyG, the Gensini score + LVEF + FBG, the Gensini score + LVEF + TG was 0.685 (*P* = 0.009), 0.650 (*P* = 0.237), 0.643 (*P* = 0.274), respectively. Only the TyG index provided a significant incremental prognostic value (cNRI = 0.238, IDI = 0.083) (Table 6, Fig. 4c).

**Table 5:** Comparative analysis of the discrimination of TyG, FBG, and TG for all-cause mortality

**Discrimination ability**	**TyG**		**FBG**		**TG**	
Sensitivity, %	76.2		66.0		59.3	
Specificity, %	46.7		46.9		53.0	
Youden’s index, %	22.9		12.3		12.9	
Accuracy, %	57.7		55.3		54	
Positive predictive value, %	45.9		42.8		42.4	
Negative predictive value, %	76.8		68.7		69.9	
C-Statistic (95% CI)	0.653 (0.589–0.717)		0.606 (0.543–0.670)		0.557 (0.493–0.620)	
Comparison	TyG VS. FBG		TyG VS. TG		FBG VS. TG	
	Difference	*P* value	Difference	*P* value	Difference	*P* value
C-Statistic	0.047	0.013	0.096	0.001	0.049	0.679
cNRI	0.255	0.007	0.300	0.007	-0.030	0.811
IDI	0.080	< 0.001	0.091	< 0.001	0.011	0.605

**Table 6:** Model performance after the addition of TyG, FBG, or TG to the basic model (Gensini score and LVEF) for predicting all-cause mortality

Model	C-Statistic	*P* value	cNRI	*P* value	IDI	*P* value
Gensini score + LVEF	0.627 (0.563–0.690)	-	-	-	-	-
Gensini score + LVEF + TyG	0.685 (0.624–0.746)	0.009	0.238	0.007	0.083	0.013
Gensini score + LVEF + FBG	0.650 (0.588–0.713)	0.237	-0.077	0.399	0.005	0.764
Gensini score + LVEF + TG	0.643 (0.581–0.705)	0.274	0.026	0.817	0.005	0.757

## Discussion

The present study was performed on the oldest-old ACS patients with DM and the follow-up period was as long as ten years. This study, for the first time, investigated the association between the TyG index and all-cause mortality in the population aged ≥ 80 years with ACS and DM. The TyG index was significantly associated with all-cause mortality in the study population regardless of whether the TyG index was used as a continuous or categorical variable. In addition, the relationship between the TyG index and all-cause mortality was dose–response. The TyG index outperformed FBG and TG in the prediction of mortality and further improved the prognostic value on the Gensini score combined with LVEF.

The mortality of coronary heart disease is increasing year by year. As the most severe clinical subtype, ACS has the poorest prognosis. Advanced age and DM are both independent risk factors for CHD, which could cause disease progression and deterioration. In addition, the pace of the population aging worldwide is accelerating, accompanied by the most rapidly growing of the oldest-old (those aged ≥ 80 years) group. It is no doubt that the oldest-old patients with ACS and DM would bring a heavy burden to social medicine and the health system. Accordingly, exploring the predictors associated with the long-term prognosis and early identifying the high-risk patients are effective strategies to deal with this problem.

The TyG index is one surrogate indicator that reflects the condition of IR in the body, which can be calculated from FBG and TG [12]. Studies have shown that the TyG index has a good correlation with traditional IR evaluation markers, such as the hyperinsulinemic-euglycemic clamp test and HOMA-IR [14, 21]. Moreover, compared with the traditional IR evaluation indicators, the TyG index could be calculated by common laboratory markers, which may be more suitable for clinical practice. Previous studies have shown that the TyG index is associated with arterial stiffness [16], coronary artery calcification progression [17, 22], and the incident of DM [22], which are all associated with the incident and progression of cardiovascular disease. In the present study, the TyG index was found to be positively correlated with eGFR, TC, LDL-C and negatively correlated with HDL-C, which are all traditional risk factors for cardiovascular disease. Based on the factors mentioned above, the hypothesis that the TyG index could predict the prognostic risk of cardiovascular disease is rational.

Further, several studies have reported that there is also a significant association between the TyG index and the risk of cardiovascular and cerebrovascular diseases. The TyG index was associated with prognostic risk in both STEMI and NSTEMI populations [23–25]. In these studies, the TyG index was found to be associated with the risk of cardiovascular and cerebrovascular events, but only one study revealed that there was a significant association between the TyG index and the risk of cardiac events and all-cause mortality. In the nondiabetic patients with ACS, the association between TyG index and mortality was not well established. Yang et al. reported that the TyG index was not associated with all-cause mortality in the nondiabetic patients undergoing PCI [26]. In another study based on the nondiabetic patients with NSTE-ACS undergoing PCI, Zhao et al. also revealed that the TyG index had no association with all-cause mortality [27]. However, Şaylık et al. found that the TyG index was associated with the in-hospital and one-year mortality in the elderly nondiabetic patients with STEMI [28]. It was clear that these studies had apparent discrepancies attributed to variations in study population, variable type of the TyG index, time of follow-up, and adjustment for confounders. So, it is need that more studies should be conducted to explore the association between the TyG and mortality in the nondiabetic patients with ACS.

In our study, given that the oldest-old patients with ACS and DM were more susceptible to the adverse outcome, the association between TyG index and all-cause mortality was investigated in this population. Besides, the relationship between the TyG index and all-cause mortality in ACS patients with DM is also not well established. There are merely four studies that have reported the association between the TyG index and all-cause mortality, and the findings of these studies were not well consistent [29–32]. Discrepancies within these studies were likely attributed to variations in the study population, variable type of the TyG index, time of follow-up, and adjustment for confounders. Of the studies mentioned above, the mean age of participants ranged from 60.9 to 66.3 years, and the time of follow-up ranged from 12 to 36 months. Different from previous studies, ACS patients with DM aged 80 years or above were enrolled and the follow-up time ranged from 36 to 113 months. It’s the first study to confirm the association between the TyG index and all-cause mortality in this special population, and also find that the association between the TyG index and mortality was dose–response. To enhance the reliability and validity of the study, we have used several statistical analysis strategies, including continuous and categorical variable types, trend test analyses, subgroup analyses and interaction analyses. Fortunately, the findings are stable and significant in different statistical analysis models. In terms of the study population and data analysis strategies, we think that our study could provide some contributions to this field.

As mentioned above, the TyG index is the product of FBG and TG. Theoretically, the TyG index represents the comprehensive effect of FBG and TG, which should be superior to either FBG or TG. The findings confirm this hypothesis. For mortality risk prediction, compared to FBG and TG, the TyG index has an advantage in the discrimination capacity. Further, the TyG index has a significant improvement in the prediction model based on the Gensini score combined with LVEF, which are the surrogate indicators of the severity of the coronary lesion and myocardial function, respectively.

The research shows that the TyG index is a dependable indicator to predict all-cause mortality in oldest-old patients with ACS and DM. Although the exact pathophysiological mechanism is not clear, we speculate that IR may be the main cause of target organ damage due to the increase of TyG index. First of all, subclinical vascular disease is associated with IR, and vascular functional and structural damage and hyperinsulinemia resulting from IR contribute to the emergence of endothelial cell dysfunction, followed by the genesis of the decrease in bioavailable nitric oxide, which leads to endothelial-dependent vascular dysfunction, including impaired vascular relaxation, inflammation, vascular remodeling, and overt fibrosis [33–37]. Secondly, IR may leads to the intensification of oxidative stress and inflammatory response, disturbance of glucose and lipid metabolism, activation of the renin–angiotensin–aldosterone system (RAAS) and thrombotic activity, and ultimately cell damage, hypertrophy, and fibrosis occur [38–40]. Furthermore, IR is closely associated with microvascular and myocardial damage and poor myocardial perfusion, which leads to various cardiovascular events [17, 29, 41].

### Comparisons with other studies and what does the current work add to the existing knowledge

This is the first study among aged 80 years or above patients with ACS and DM. In this special population, the findings of this study have acknowledged the relationship between the TyG index and all-cause mortality and the dose–response relationship between the TyG index and mortality.

### Study strengths and limitations

The present study has a number of strengths. The current study has confirmed the TyG index was associated with all-cause mortality in oldest-old ACS patients with DM. And the TyG index outperformed FBG and TG in the prediction of mortality. The TyG index improved the prognostic value on the Gensini score combined with LVEF. There are some limitations in the present study. Firstly, this study is a single-center study, and the sample size is relatively small. There is a certain selection bias, and further multi-center and large-sample studies are needed to verify these findings. Secondly, this study did not conduct a comparative analysis between the TyG index and traditional IR indicators. Thirdly, although many confounding factors have been included in the research analysis, there are still some known factors (dietary habits and nutritional status) or unknown factors that may affect the results that have not been included in the analysis.

## Conclusions

The TyG index predicts the risk of all-cause mortality in the oldest-old ACS patients with DM. Particularly, the TyG index is better than FBG and TG in the prediction of mortality risk and improves the prognostic value of the Gensini score combined with LVEF. The TyG index may become a convenient biomarker for screening and predicting all-cause mortality in the oldest-old ACS patients with DM in the future.

.

## Data Availability

The datasets used and/or analyzed during the current study are available from the corresponding author on reasonable request.

## Abbreviations

ACS: Acute coronary syndrome
DM: Diabetes mellitus
IR: Insulin resistance
CHD: Coronary heart disease
UAP: Unstable angina pectoris
NSTEMI: Non-ST-segment elevation myocardial infarction
STEMI: ST-segment elevation myocardial infarction
BMI: Body mass index
HR: Heart rate
SBP: Systolic blood pressure
DBP: Diastolic blood pressure
LVEF: Left ventricular ejection fraction
eGFR: Estimated glomerular fltration rate
FBG: Fasting blood glucose
UA: Uric acid
TC: Total cholesterol
TG: Triglyceride
LDL-C: Low-density lipoprotein-C
HDL-C: High-density lipoprotein-C
TyG: Triglyceride glucose
MI: Myocardial infarction
CKD: Chronic kidney disease
ACEI: Angiotensin-converting enzyme inhibitor
ARB: Angiotensin receptor blocker
LAD: Left anterior descending artery
LCX: Left circumfex artery
RCA: Right coronary artery
LM: Left main coronary artery
PCI: Percutaneous coronary intervention
CABG: Coronary artery bypass grafting
HR: Hazard ratio
CI: Confidence interval
SD: Standard deviation
ROC: Receiver operating characteristic
cNRI: Continuous net reclassification improvement
IDI: Integrated discrimination improvement
